# Microstructural Constituents and Mechanical Properties of Low-Density Fe-Cr-Ni-Mn-Al-C Stainless Steels

**DOI:** 10.3390/ma15155121

**Published:** 2022-07-23

**Authors:** Steffen Scherbring, Guanghui Chen, Bastian Veltel, Gert Bartzsch, Julia Richter, Malte Vollmer, Malte Blankenburg, Saikat Shyamal, Olena Volkova, Thomas Niendorf, Ulrich Lienert, Puspendu Sahu, Javad Mola

**Affiliations:** 1 Materials Design and Structural Integrity Laboratory, Faculty of Engineering and Computer Sciences, Osnabrück University of Applied Sciences, 49076 Osnabrück, Germany; steffen.scherbring@hs-osnabrueck.de (S.S.); chenguanghui@wust.edu.cn (G.C.); baveltel@gmail.com (B.V.); 2 Institute of Iron and Steel Technology, Technische Universität Bergakademie Freiberg, 09599 Freiberg, Germany; gert.bartzsch@iest.tu-freiberg.de (G.B.); volkova@iest.tu-freiberg.de (O.V.); 3 Institute of Materials Engineering—Metallic Materials, University of Kassel, 34125 Kassel, Germany; julia.richter@uni-kassel.de (J.R.); vollmer@uni-kassel.de (M.V.); niendorf@uni-kassel.de (T.N.); 4 Deutsches Elektronen-Synchrotron (DESY), Photon Science, 22607 Hamburg, Germany; malte.blankenburg@desy.de (M.B.); ulrich.lienert@desy.de (U.L.); 5 Department of Physics, Jadavpur University, Kolkata 700032, India; saikat.shyamal923@gmail.com (S.S.); psahu74@gmail.com (P.S.)

**Keywords:** low-density steel, stainless steel, intermetallics, mechanical properties, microstructure

## Abstract

Metallic material concepts associated with the sustainable and efficient use of resources are currently the subject of intensive research. Al addition to steel offers advantages in view of lightweight, durability, and efficient use of high-Fe scrap from the Al industry. In the present work, Al was added to Fe-12Cr-(9,12)Ni-3Mn-0.3C-xAl (x = 0.1–6) (wt.%) stainless steels to assess its influence on microstructure and mechanical properties. According to density measurements based on Archimedes’ principle, densities were between 7.70 and 7.08 g/cm^3^. High-energy X-ray diffraction estimations of the lattice parameter indicated that nearly 31% of density reduction was caused by the lattice expansion associated with Al addition. Depending on Al concentration, austenitic and duplex matrix microstructures were obtained at room temperature. In the presence of up to 3 wt.% Al, the microstructure remained austenitic. At the same time, strength and hardness were slightly enhanced. Al addition in higher quantities resulted in the formation of duplex matrix microstructures with enhanced yield strength but reduced ductility compared to the austenitic alloys. Due to the ready formation of B2-(Ni,Fe)Al intermetallics in the ferrite phase of the present alloy system, the increase in strength due to the presence of ferrite was more pronounced compared to standard duplex stainless steels. The occurrence of B2 intermetallics was implied by dilatometry measurements and confirmed by electron microscopy examinations and high-energy X-ray diffraction measurements.

## 1. Introduction

In order to preserve the environment and guarantee resource efficiency, it is important to intensify efforts on sustainable material solutions. A well-established strategy involves increasing the specific strength by microstructure design aiming for a raised yield strength [1]. Another approach is to reduce the density. Al addition to steels offers the potential to combine both approaches [2,3,4]. Due to their high corrosion resistance and durability, stainless steels are among sustainable material solutions [5,6]. They can also be reused in a circular economy [7,8]. Stainless steels with high Al contents have been developed for reasons such as raised corrosion resistance and enhanced oxidation performance at high temperatures [2,9,10,11]. Given that the majority of conventional stainless steels are austenitic and therefore paramagnetic, it is not possible to use scrap-lifting magnets to separate them from non-magnetic scraps, such as Al components. Design of Al-alloyed stainless steels can increase the tolerance to Al-containing stainless-steel scrap, since a complete separation is then no longer necessary. Furthermore, it provides a solution to exploit low-grade Al scrap, for instance, scrap containing a high Fe content, which is not recyclable without extensive metallurgical purification.

Although the concept of Al addition to stainless steels is very promising in view of resource efficiency, it cannot be a viable solution unless the associated metallurgical changes in both liquid and solid states, as well as interactions of Al with Fe and alloying elements, are closely examined. In the solid state, alloying elements such as C, Cr, Ni, Mn, Mo and N are commonly used to control the microstructure and mechanical properties of stainless steels [12,13]. By increasing the thermodynamic driving force for the formation of ferrite, Al contributes to the stabilization of the ferrite phase. Accordingly, if Al is gradually added to an austenitic stainless steel, the base microstructure changes to austenitic-ferritic (duplex) [14]. Duplex stainless steels exhibit a favorable combination of ductility and strength, imparted by austenite and ferrite, respectively [15]. In other words, the presence of ferrite in duplex stainless steel is responsible for an increased yield strength and hardness compared to austenitic counterparts [2,13,16]. In the presence of even higher Al contents, fully ferritic matrix microstructures are obtained [14,17,18,19,20,21]. Al-alloyed stainless steels could even exhibit martensitic matrix microstructures if the following two conditions are met. On the one hand, high-temperature austenite must be guaranteed by maintaining a high ratio of Ni-equivalent to Cr-equivalent. This limits the permissible Al concentration. On the other hand, a high martensite start temperature must be adjusted by a relatively lean alloy design [22].

To counteract the ferrite-stabilizing effect of Al, the austenite potential can be raised by the addition of elements such as C, Ni and Mn. By an appropriate balance of alloying elements, it is also possible to obtain Al-added steels with fully austenitic microstructures. Al addition to Hadfield steels and stainless steels with austenitic microstructures has been found to improve the wear resistance and resistance to hydrogen embrittlement [13,23,24,25].

Compared to ferrite, the work-hardening behavior of austenite is subject to considerable variations depending on the deformation mechanism. The deformation mechanism is, in turn, regulated by the stacking fault energy (SFE). The SFE is largely affected by temperature and chemical composition [26,27,28]. In particular, Al is known to increase the SFE [29,30]. To account for the influence of dislocation density and character on the strain energy of stacking faults, the concept of “effective SFE” has been proposed [31]. At SFEs below nearly 20 mJ/m^2^, the deformation mode of austenite is dominated by deformation-induced martensite formation. SFEs between 20–40 mJ/m^2^ have been suggested to be favorable for the occurrence of deformation twinning. As the SFE increases further (SFE > 40 mJ/m^2^), partial dislocations can constrict and cross slip. As a result, glide planarity is gradually reduced. This results in wavy glide and the formation of dislocation cells [26,27].

In addition to its influence on the SFE, Al addition to austenitic stainless steel could change the solidification mode from austenitic to ferritic [4,32]. Due to differences in the segregation behavior of alloying elements between dendritic and interdendritic areas in cases of austenitic and ferritic solidification [33,34,35,36], the spatial distribution of alloying elements such as Al, Cr, Mn and Ni—therefore, the spatial distribution of SFE—will be influenced if the solidification mode is changed.

In the case of Al-added stainless steels with ferritic matrix microstructures, the formation of B2 intermetallics is known to occur, especially if nickel is also present [17,18,19]. The orientation relationship between the B2 phase and ferrite is cube-on-cube, namely, (011)_B2_//(011)_α_ and [0,1,2,3,4,5,6,7,8,9,10,11]_B2_//[0,1,2,3,4,5,6,7,8,9,10,11]_α_. B2 formation in Al-added martensitic stainless steels can lead to very high strength levels, as exemplified by Fe-10.5Cr-12Ni-3Al [22]. Hardness levels in excess of 600 HV in B2-strengthened martensitic stainless steels make them, strength-wise, superior to 17-4PH and 15-5PH precipitation-hardenable stainless steels and comparable to 18Ni maraging steels.

The present work includes a detailed investigation of the microstructure and mechanical properties in novel Fe-Cr-Ni-Mn-Al-C stainless steels with varying contents of Al. Compared to our first developments [37,38], C and Cr concentrations are reduced to oppose the formation of Cr-rich carbides. Compared to the second generation [4], which were already low in C and Cr but mechanically too stable to exhibit maximum elongation at room temperature, a leaner alloy design is proposed and investigated here. Therefore, this study can be regarded as a consecutive exploration of Al-alloyed steels. The microstructural characterizations and mechanical tests conducted in the present work aim to clarify the microstructure–property relationship in this novel class of stainless steels and pave the way for further optimizations.

## 2. Materials and Experimental Procedure

Experimental materials had nominal compositions Fe-12Cr-9Ni-3Mn-xAl-0.3C (wt.%) with x varying between nearly 0.1 and 6 wt.%, in increments of approximately 1.5 wt.%. To examine the effect of Ni, one additional alloy containing nearly 6 wt.% Al and a raised Ni concentration of 12 wt.% was cast as well. The alloy IDs and chemical compositions are given in Table 1. The Al content was quantified by inductively coupled plasma optical emission spectroscopy (ICP-OES) using an Agilent 5100 VDV spectrometer. The C content, on the other hand, was determined by combustion analysis using Bruker G4 ICARUS equipment. The remainder of the elements were quantified by optical emission spectrometry using a Belek Vario Lab spectrometer. Ingots were prepared by melting and casting in a vacuum induction melting (VIM) furnace under an Ar atmosphere. To adjust the chemical compositions according to Table 1, Armco iron and high-purity elements were added to a Fe-17Cr-0.05C master alloy. Cast ingots were homogenized at 1150 °C for 1 h and subsequently cooled with an approximate cooling rate of 50 K/s. The density was measured in accordance with Archimedes’ principle by measuring the buoyant force (upthrust). A Kern ABT01 density measurement system equipped with a Kern ABT 220 precision scale was used for the measurements [39].

For the examination of undeformed microstructures, specimens in the as-homogenized condition were prepared by electrical discharge machining (wire cutting). To eliminate the influence of wire cutting, specimens were ground with 1200 and 2000 grit SiC papers under a stream of boiling water. This was followed by multiple polishing steps with a mixture of diamond paste (9, 3 and 1 µm-particles) and lubricant for a mirror-like surface finish. For the final polish, 24 h of vibration polishing with 0.05 µm colloidal suspension was used.

Microstructure characterizations using secondary electrons (SE), electron channeling contrast imaging (ECCI) and electron backscatter diffraction (EBSD) methods were performed in Zeiss Auriga scanning electron microscope (SEM). 

Vickers hardness measurements with an indentation load of 10 kgf (HV10) were performed according to the DIN EN ISO 6507-1 standard using a Wolpert universal hardness tester [40]. Quasi-static tensile tests with an initial strain rate of 10^−4^ s^−1^ were conducted using a Zwick Kappa 100 DS machine. Round tensile specimens with threaded shoulders (M10) were machined from materials in the as-homogenized condition in accordance with the DIN 50125 standard [41]. The parallel length, gage length and gage diameter were 36 mm, 30 mm and 6 mm, respectively. To eliminate the mechanical damage induced near the surface by machining, machined tensile specimens were solution annealed at 1150 °C for 5 min under an Ar atmosphere and cooled with an air gun. In this manner, an estimated average cooling rate of 30 K/s was obtained between 1150 °C and 600 °C.

Dilatometry studies using cylindrical rods, 10 mm in diameter and 15 mm in length, were carried out using the dilatometry module of a Gleeble 3500 GTC thermomechanical simulator. Heating and cooling were performed under a vacuum. The heating and cooling rates were both 5 K/s. The apparent coefficient of thermal expansion (CTE_a_) was determined using the first derivative of relative length change with respect to temperature. From the temperature of interest, a range of −15 °C to +15 °C was used to construct the first derivative curve. This procedure was intended to reduce CTE_a_ fluctuations that would otherwise occur if derivation was performed using two consecutive data points. Due to conductive heating in the Gleeble dilatometer, heating-rate-dependent irregularities arising from specimen–inductor interactions and the abrupt release of magnetostrictive forces encountered in dilatometers with inductive heating were avoided [17,42]. To study the solidification mode, differential scanning calorimetry (DSC) measurements were performed under vacuum in a Netzsch-404C Pegasus calorimeter. For the measurements, cylindrical specimens with a diameter of 4 mm and a height of 1 mm of all alloys were heated to 1550 °C at 50 K/min and then cooled to room temperature at 10 K/min. To ensure reproducibility, 2 specimens were measured from each alloy.

**Figure 1:**
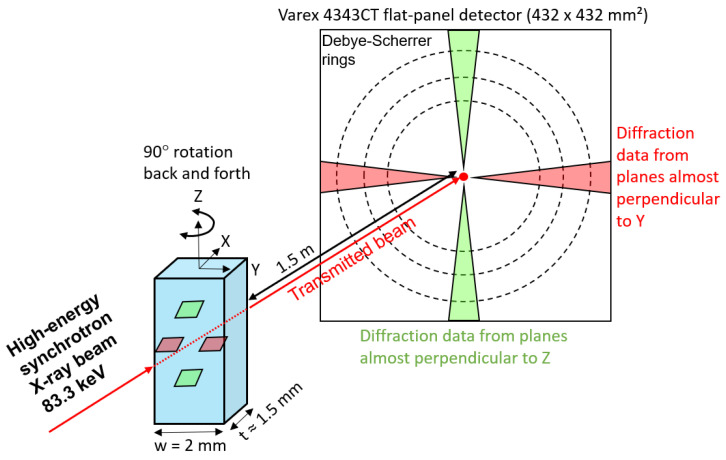
Schematic illustration of experimental setup for the HE-XRD experiments at DESY.

Diffraction measurements with a high-energy monochromatic X-ray beam (HE-XRD) having a photon energy of 83.3 keV, corresponding to a wavelength of 0.14884 Å, were performed at the PETRA III, P21.2 beamline at the “Deutsches Elektronen-Synchrotron”, (DESY). The schematic illustration of the experimental setup is shown in Figure 1. The beam size was controlled by slits to an area 200 × 200 µm^2^ in size. This was carried out to obtain a high diffracted signal intensity while covering a relatively large number of grains. A Varex 4343CT flat-panel detector with 2880 × 2880 pixels, each 150 × 150 µm^2^ in size, was positioned about 1.5 m farther from the specimen. The specimens used for the HE-XRD experiments were obtained by further processing of the as-homogenized material for the purpose of grain refinement. Grain refinement reduces the spottiness of diffraction patterns by increasing the number of grains meeting the Bragg condition. Processing for grain refinement involved double-step cold rolling with intermediate and final recrystallization annealing at 1150 °C for 5 min. Since the HE-XRD experiments were continued after applying different tensile strain levels (not relevant to the present study), the 6Al steel with poor ductility was not investigated.

For each experiment, the specimen was subjected to a back-and-forth rotation around the *z*-axis to 90° with an angular speed of 5° per second, while pausing for 1 s at 90°. Then, 2D diffraction patterns were recorded continuously with an exposure time of 1 s. In this manner, a total of 38 diffraction patterns were collected for each specimen. These patterns were subsequently superimposed to reduce the spottiness of diffraction patterns caused by the relatively large grain size of experimental steels compared to the volume exposed to the X-ray beam. The 2D diffraction patterns were then azimuthally integrated (0–360°) using the PyFAI software [43,44] to obtain an intensity vs. 2θ ASCII format data. The diffraction patterns were further calibrated for the angular positions using a LaB6 specimen supplied by the National Institute of Standards and Technology (NIST). The 1D diffraction patterns were then analyzed to determine the lattice parameter. The X-ray profile shapes were approximated by a pseudo-Voigt function.

## 3. Results and Discussion

### 3.1. Thermodynamic Equilibrium Calculations and Solidification Mode

**Figure 2:**
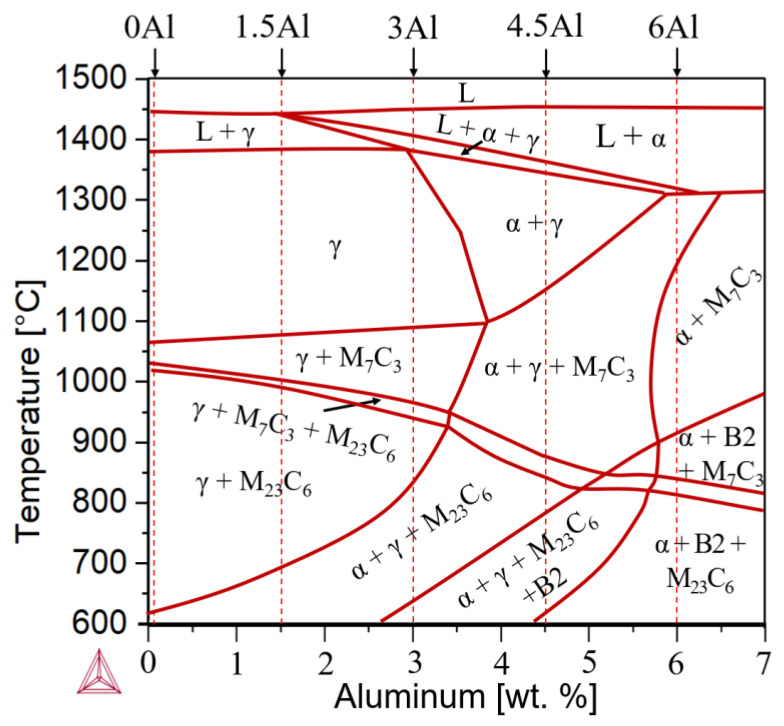
Pseudo-binary phase diagram of Fe-12Cr-9Ni-3Mn-0.3C as a function of Al content, calculated using Thermo-Calc. The positions of alloys are marked by vertical dashed lines.

Figure 2 shows the pseudo-binary Fe-12Cr-9Ni-3Mn-0.3C-xAl (x = 0–7 wt.%) phase diagram calculated by the TCFE9 database of Thermo-Calc. The calculated diagram suggests that the equilibrium matrix microstructure at 600 °C consists of ferrite and austenite for 0Al, 1.5Al and 3Al steels but only of ferrite for 4.5Al and 6Al steels. Furthermore, C is stabilized in the form of M_23_C_6_ carbides. For 3Al, 4.5Al and 6Al steels, the presence of the B2 phase is also predicted by Thermo-Calc. The type of carbides changes to M_7_C_3_ at higher temperatures. The phase diagram also suggests that the solidification of all steels containing 9 wt.% Ni, except for 0Al with an entirely austenitic (A) solidification mode, begins with the formation of ferrite and continues with the formation of austenite (FA solidification mode). The formation of austenite in the 1.5Al steel begins slightly below liquidus temperature, whereas it is postponed to the latest stages of solidification, namely, slightly above solidus temperature, in the case of 6Al. An overview of solidification modes based on Thermo-Calc calculations is provided in Table 2.

**Figure 3:**
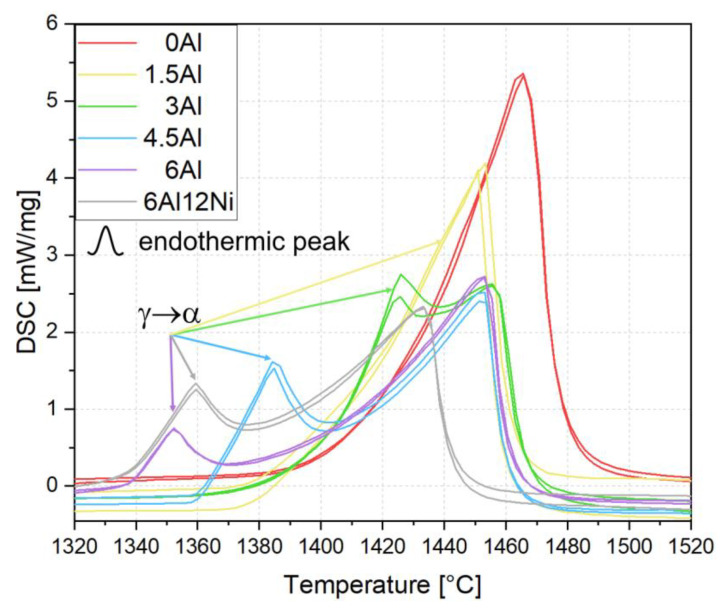
DSC results during heating up to 1520 °C for the interpretation of the sequence of transformations during solidification.

To examine the Thermo-Calc predictions with regard to the solidification mode, heat-exchange curves during the heating segment of DSC measurements are shown in Figure 3. The use of the DSC signal during heating for the interpretation of reactions during the preceding solidification offers numerous advantages including reduced contamination, reduced evaporation of alloying elements as well as a high contact area with the bottom of the DSC crucible [22]. This leads to highly reproducible DSC data, as evidenced by the close proximity of the two DSC curves for each specimen. According to the DSC traces in Figure 3, the melting of the 0Al alloy is associated with a single endothermic peak, indicating the absence of ferrite formation, thereby confirming the A solidification mode as predicted by Thermo-Calc. For the remainder of the alloys, melting occurs with two endothermic peaks, the lower temperature peak being related to the formation of ferrite. In the case of 1.5Al, the ferrite formation peak is less noticeable than alloys with higher Al contents. The ferrite formed from austenite during heating would then melt at higher temperatures. Given that the solidification of 1.5Al begins with the formation of ferrite and the stability of ferrite relative to austenite increases at higher Al contents, the solidification of all alloys except 0Al begins with the formation of ferrite. Accordingly, the onset of the ferrite formation peak during heating is shifted to lower temperatures as the Al concentration is raised. Since the peak due to the formation of ferrite overlaps with the peak due to the melting of ferrite, even in the case of 6Al, an FA solidification mode is expected for all alloys except 0Al (Table 2). This agrees with the Thermo-Calc prediction results.

Variations in the intensity of the ferrite formation peak during heating could be justified by the already-existing ferrite in the solid state. Accordingly, the lower intensity of the γ → α peak in the 6Al steel compared to that for the 6Al12Ni steel implies the presence of a higher fraction of ferrite in the 6Al steel just below the solidus temperature and agrees well with its lower Ni content. The observations made here with regard to the effect of Al on the solidification mode are in good agreement with the DSC results reported in the authors’ previous studies [4,32].

### 3.2. Contributions to Density Reduction

**Figure 4:**
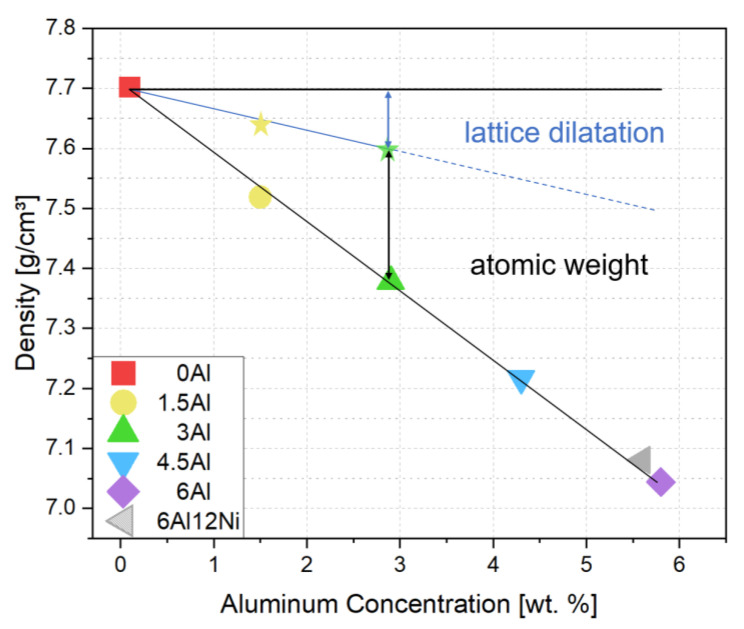
Density as a function of Al content. Hatched fill for the 6Al12Ni alloy is meant to emphasize its higher Ni content compared to the rest of the alloys. For the austenitic alloys, namely, alloys with up to 3 wt.% Al, the contributions of lattice dilatation and atomic weight to density reduction are separated by the solid line passing through star symbols. Star symbols indicate density on the mere basis of lattice parameter change with respect to the 0Al steel. The dashed line for duplex alloys indicates that the separation of contributions to density reduction was not based on actual lattice parameter measurements but extrapolated from the range of austenitic alloys.

**Figure 5:**
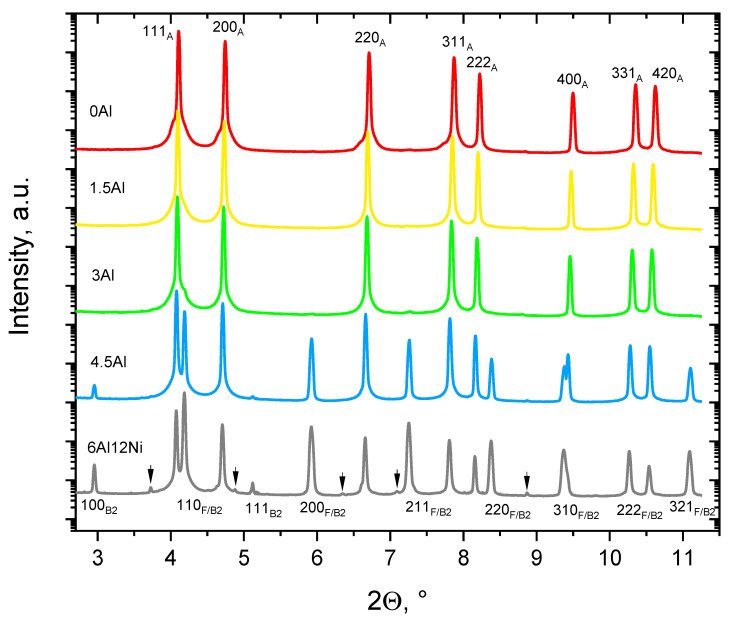
1D XRD patterns of experimental steels obtained by post-processing of 2D diffraction patterns. Specimens were obtained by homogenization, double-step cold rolling and recrystallization annealing of cast ingots. Arrows indicate weak reflections likely due to hexagonal M_7_C_3_ carbides.

Density measurements indicated a linear relationship between the density and Al concentration, as shown in Figure 4. The experimental steels show a weight reduction potential of 1.45% per 1 wt.% of Al addition. This is comparable with the density reduction of 1.3% per 1 wt.% Al reported for an Al-alloyed Hadfield steel by Frommeyer et al. [15]. Lattice expansion and the lower atomic weight of Al compared to the common substitutional alloying elements in steels are the two mechanisms responsible for the density reduction upon Al addition to steels [45,46,47]. To quantify the contribution of lattice expansion to density reduction in the present alloys, lattice parameters were measured by HE-XRD. The 1D diffraction patterns in Figure 5 indicate almost fully austenitic microstructures for 0Al, 1.5Al and 3Al steels. A closer inspection of diffraction patterns in Figure 5 indicates weak peaks attributable to ferrite or martensite in 0Al, 1.5Al and 3Al steels, which have formed close to the surface due to decarburization during heat treatment or due to the mechanical damage induced by grinding.

Due to the negligibility of phases other than austenite, thus the irrelevance of lattice parameter changes due to the partitioning of alloying elements among phases, the lattice parameters of 0Al, 1.5Al, and 3Al steels were used to quantify the lattice dilatation caused by Al. The austenite lattice parameters in 0Al, 1.5Al and 3Al steels were calculated to be 0.35964 ± 0.8 × 10^−4^ nm, 0.36059 ± 1.0 × 10^−4^ nm and 0.36127 ± 0.7 × 10^−4^ nm, respectively. On this basis, the addition of each wt.% Al results in an increase in the austenite lattice parameter by nearly 5.4 × 10^−4^ nm, which agrees well with the authors’ previous studies [4,48] as well as with the results reported in [49]. Accordingly, as marked in Figure 4 by star symbols and the solid line passing through them, density reduction caused merely by lattice expansion of austenite accounts for nearly 31% of the total density reduction. Since the density of steels continues to decrease almost linearly at higher Al concentrations and the share of density reduction due to the substitution of atoms by Al remains constant, the share of density reduction due to lattice expansion is retained at Al concentrations above 3 wt.%, where the microstructures are no longer fully austenitic.

### 3.3. Microstructural Aspects 

**Figure 6:**
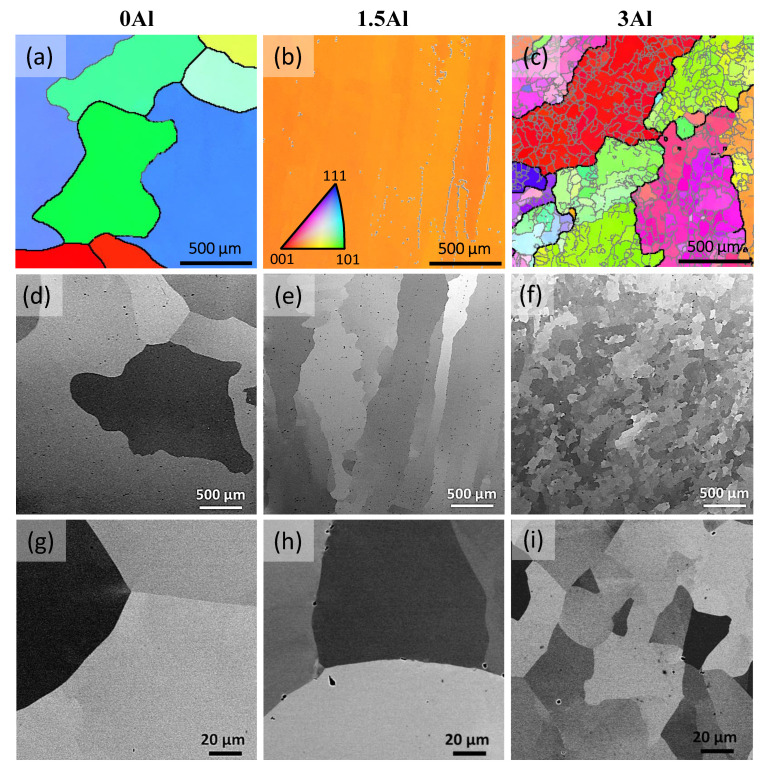
EBSD IPF maps (**a**–**c**), and SEM micrographs (**d**–**i**) for the austenitic steels in the homogenized condition; (**a**,**d**,**g**) 0Al, (**b**,**e**,**h**) 1.5Al and (**c**,**f**,**i**) 3Al steels. Black and gray lines superimposed on IPF maps indicate HAGBs and LAGBs, respectively. HAGBs and LAGBs refer to boundaries with misorientation angles above 15° and in the range of 1–15°, respectively. The lower threshold for LAGBs was reduced to 0.5° for the 1.5Al steel.

Figure 6 presents the SEM results for 0Al, 1.5Al and 3Al steels in the homogenized condition. According to the EBSD inverse pole figure (IPF) maps for austenite in Figure 6a–c, the microstructure of all steels consists of coarse austenite grains of the order of a few hundred microns. In addition, subgrain boundaries characterized by small misorientation angles can be observed in 1.5Al and 3Al steels, especially in the latter steel. Subgrain boundaries are highlighted by superimposition of low-angle grain boundaries (LAGBs) on the EBSD IPF maps. The abundance of subgrains in the 3Al steel, their occasional observation in the 1.5Al steel, and their absence in the 0Al steel are also revealed in the SEM micrographs of Figure 6d–i. The occurrence of subgrains in Fe-18Cr-9Ni-7Mn-4Al-0.43C steel has been explained by the formation of ferrite during solidification [32]. Based on Thermo-Calc predictions and DSC results presented in Section 3.1, this explanation is also applicable to present steels. Regions containing subgrains in the 1.5Al steel are expected to correspond to those solidified as ferrite.

**Figure 7:**
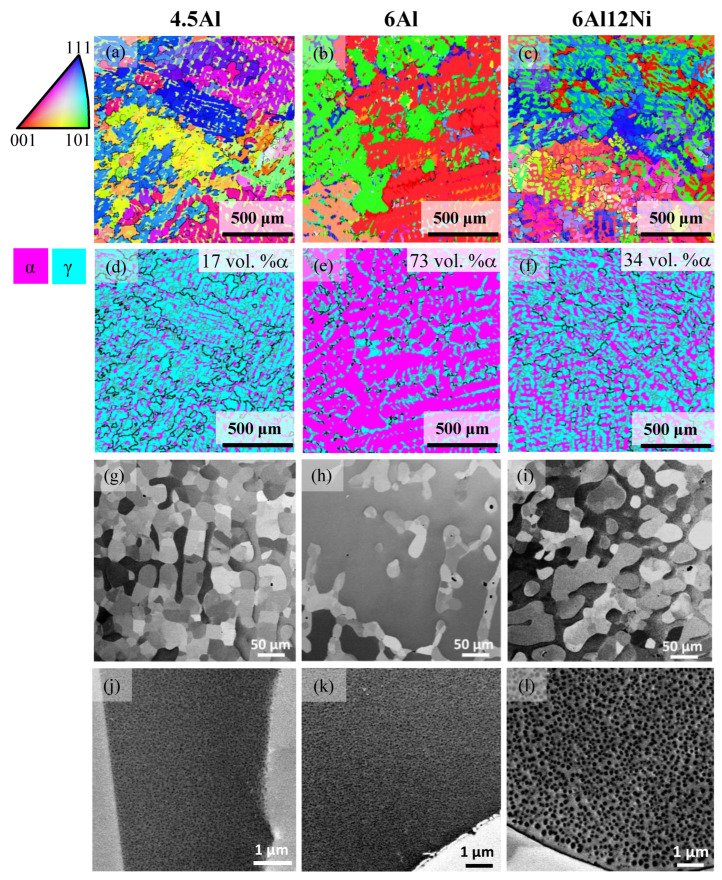
EBSD IPF maps (**a**–**c**), EBSD phase maps (**d**–**f**), and SEM micrographs (**g**–**l**) for the homogenized steels with duplex matrix microstructures; (**a**,**d**,**g**,**j**) 4.5Al steel, (**b**,**e**,**h**,**k**) 6Al steel and (**c**,**f**,**i**,**l**) 6Al12Ni steels. The SEM micrographs in (**j**–**l**) provide a magnified view of features in ferrite. Black and gray lines superimposed on EBSD phase maps indicate HAGBs (misorientation angles larger than 15°) and LAGBs (misorientation angles in the range of 1–15°) within each phase, respectively.

Figure 7 presents the SEM results for 4.5Al, 6Al and 6Al12Ni steels in the as-homogenized condition. According to the EBSD IPF maps in Figure 7a–c and the corresponding phase maps in Figure 7d–f, duplex ferritic–austenitic matrix microstructures are identified in all cases. The ferrite fractions quantified by EBSD for 4.5Al, 6Al and 6Al12Ni steels were 17, 73, and 34 vol.%, respectively. These fractions are in qualitative agreement with the relative Al and Ni concentrations of these alloys. Given the ferritic solidification of duplex alloys, the ferritic regions persisting in the room temperature microstructure correspond to the dendrite cores. This is due to the inverse segregation of Al in the case of ferritic solidification, namely, its enrichment within the solid dendrites and its depletion in the coexisting liquid phase. The remainder of alloying elements, on the other hand, enrich in the coexisting liquid phase. This segregation pattern increases the austenite potential of the liquid phase and favors the formation of austenite at later stages of solidification [33,34,35,36,50]. Due to the involvement of ferrite as a precursor of austenite, austenitic regions in the duplex alloys contain subgrains. The subgrains are best revealed by the LAGBs superimposed on the EBSD phases maps in Figure 7d–f and contrast variations in the SEM images of Figure 7g–i.

The SEM micrographs in Figure 7j–l provide a magnified view of the ferrite phase in the duplex alloys. Contrast variations within ferrite in 4.5Al and 6Al alloys (Figure 7j–k) imply the presence of precipitates within ferrite. The presence of an array of precipitates is most obvious for the 6Al12Ni steel (Figure 7l). To examine the possibility of precipitates within ferrite being B2-(Ni,Fe)Al intermetallics, as also predicted by Thermo-Calc, the HE-XRD patterns for 4.5Al and 6Al12Ni steels were consulted (Figure 5). According to the patterns, ferrite peaks are readily detected in the 4.5Al and 6Al12Ni steels. Due to the coincidence of the majority of peaks for the B2-(Ni,Fe)Al intermetallics and ferrite, they are reported not to be distinguished readily [22]. However, the characteristic (100)_B2_ and (111)_B2_ peaks [29,51] are readily identified in the diffraction patterns for 4.5Al and 6Al12Ni steels, especially in the latter alloy. This reaffirms that the presence of ferrite and a high concentration of Al and Ni both promote the formation of B2. As marked by arrows in Figure 5, weak reflections not attributable to austenite, ferrite, and B2 were identified for the 6Al12Ni steel. Peak positions are in fair agreement with those for hexagonal M_7_C_3_ [52,53]. Nevertheless, due to the small number of peaks and the existence of two perspectives (orthorhombic and hexagonal) for the crystal structure of M_7_C_3_ [37,54], a reliable phase identification was not possible. The facilitated occurrence of carbides in duplex steels is justified by the low solubility of C in ferrite, thereby its enrichment and precipitation as carbide in the coexisting austenite.

**Figure 8:**
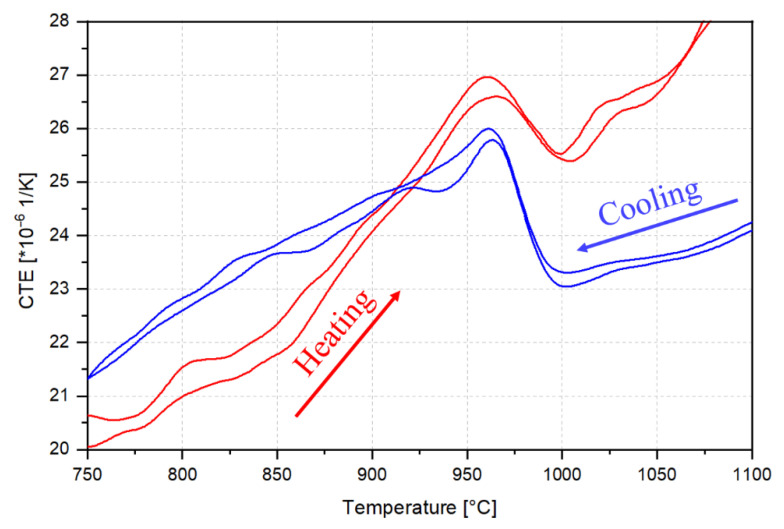
Representative CTE_a_ curves as functions of temperature during heating and cooling of the 6Al steel at 5 K/s.

Dilatometry was used to study the dissolution and precipitation of B2 intermetallics in the 6Al steel with the highest fraction of ferrite, therefore the highest ability to form B2. This would amplify the length change signal due to the evolution of B2 within ferrite. Based on the diameter change with the temperature of a single specimen during sequential heating to 1250 °C at a rate of 5 K/s and cooling at the same rate, CTE_a_ values were calculated as functions of temperature. Two representative CTE_a_ curves during heating and two curves during cooling are plotted in Figure 8. In the absence of phase transformations and elemental redistribution among phases, the CTE_a_ would correspond to the rule-of-mixture CTE of individual phases. In the present case, however, the possible evolution of ferrite and austenite fractions with temperature and possible changes in the fraction of precipitates such as Cr-rich carbides (e.g., interphase boundary carbides, as in Figure 7k) and B2 intermetallics overlap with the base rule-of-mixture CTE of the alloy. Therefore, dynamic microstructure evolution is a source of complication during the interpretation of dilatometry data. Nevertheless, the CTE_a_ signal is very useful for the interpretation of highly reversible transformations. The high reversibility of B2 dissolution and formation during heating and cooling of a ferritic Fe-17Cr-9Ni-7Al-6Mn-0.46C (wt.%) stainless steel has been demonstrated in [17], for which the onset of B2 formation during cooling and the end of dissolution during heating occurred over a narrow temperature range between 1025 °C and 1050 °C. The net expansion during the dissolution of B2-(Ni,Fe)Al intermetallics has been shown to be mainly related to the Al and Ni enrichment of ferrite, thus an expanded ferrite lattice [17].

According to Figure 8, abrupt CTE_a_ changes occur during thermal cycling of the 6Al alloy in the vicinity of 975 °C. By analogy with the Fe-17Cr-9Ni-7Al-6Mn-0.46C steel [17], the CTE_a_ increase starting at around 850 °C during heating of the 6Al steel can be attributed to the dissolution of B2 precipitates. The CTE_a_ decreases abruptly at the approximate temperature of 960–1000 °C (Figure 8), which is an indication of an approach towards the end of B2 dissolution. The B2 dissolution-finish temperature of 1000 °C in the present case is in qualitative agreement with the B2 dissolution-finish temperature of 1050 °C for the Fe-17Cr-9Ni-7Al-6Mn-0.46C ferritic steel with a higher Al content compared to the 6Al steel in the present work [17]. However, it is somewhat higher than the Thermo-Calc prediction of 920 °C (Figure 2). During cooling, the increase in the CTE_a_ at nearly 1000 °C would indicate a net contraction as B2 begins to form. This contraction could be justified by the depletion of ferrite with respect to the constituents of B2, in particular Al. Due to the possibility of dynamic changes in the balance of phases during the dilatometry heating cycles with the multiphase 6Al steel, no effort was made to interpret the progression of dilatometry curves over the entire temperature range.

### 3.4. Hardness

**Figure 9:**
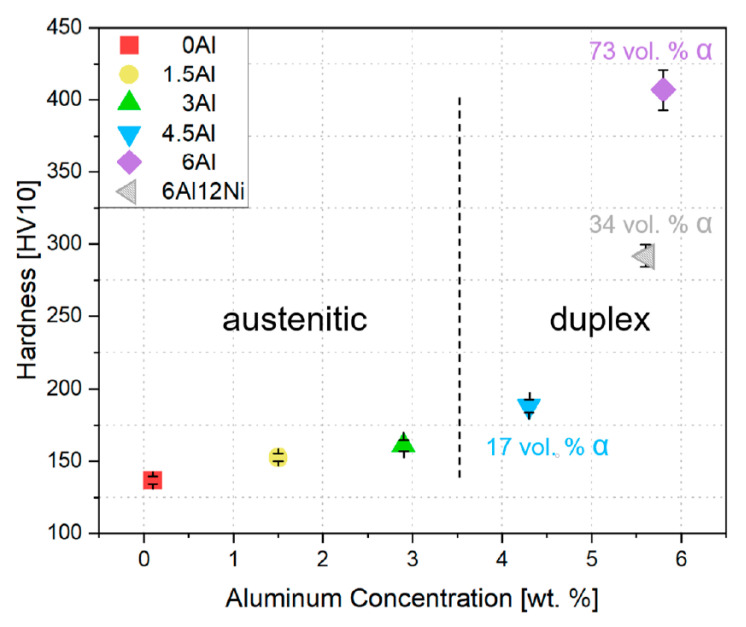
Vickers hardness as a function of Al concentration. Hatched fill for the 6Al12Ni alloy is meant to emphasize its higher Ni content compared to the rest of the alloys. Ferrite fractions marked for the duplex steels are EBSD-based.

The Vickers hardness of specimens in the homogenized condition was measured with an indentation load of 10 kgf. According to the hardness values presented in Figure 9, hardness for the austenitic steels 0Al, 1.5Al and 3Al increases slightly with Al content. This indicates a solid solution strengthening effect of Al. Although subgrain boundaries are not as efficient as high-angle grain boundaries against the slip transmission [14], strengthening by subgrain boundaries might have contributed partially to the higher hardness of 1.5Al and 3Al steels. Since hardness measurements involve significant plastic deformation of regions near the indenter, differences in deformation-induced processes, thus differences in the work hardening rate, also play a role. The work hardening behavior of steels under tensile loading conditions is discussed in Section 3.5.

For the duplex steels 4.5Al and 6Al, the hardness increases further as the Al content increases. The higher hardness of 4.5Al steel compared with the 3Al steel results from the solid solution strengthening of Al and the presence of ferrite, which is additionally strengthened by the precipitation of B2 intermetallics. The hardness increases significantly as the Al concentration increases to 6 wt.%. On the one hand, this is related to the increase in the ferrite fraction. On the other hand, the driving force for the formation of B2-(Ni,Fe)Al intermetallics in ferrite increases with Al concentration [18,55]. The strengthening contribution of B2-(Ni,Fe)Al intermetallics in steels with ferritic [14] and martensitic [22,56] matrix microstructures is well-documented. In the case of a Fe-10.5Cr-12Ni-3Al martensitic stainless steel, strengthening by B2 after a short aging treatment resulted in hardness values in excess of 600 HV [22], which exceeds the hardness of standard precipitation-hardenable stainless steels such as 15-5PH and 17-4PH, in which Cu precipitates are responsible for precipitation hardening [57,58,59]. The lower hardness of 6Al12Ni steel compared to 6Al steel—despite the higher driving force for the formation of B2 in the former steel—can be attributed to the reduction in the fraction of ferrite.

### 3.5. Tensile Properties, Work Hardening Behavior and Deformation-Induced Processes

**Figure 10:**
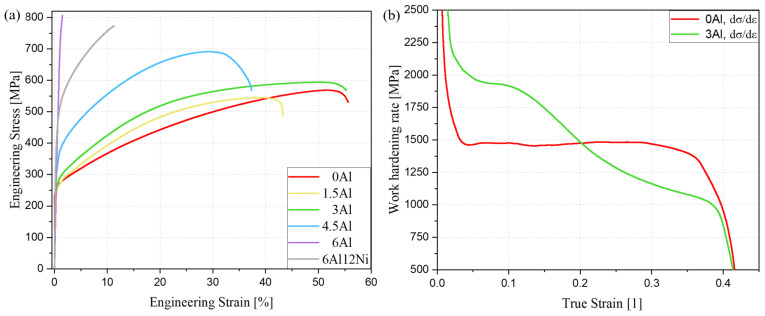
(**a**) Engineering stress–strain curves for all steels; (**b**) true work hardening rates for 0Al and 3Al steels with σ and ε denoting true stress and true strain, respectively.

Figure 10a presents the engineering stress–strain curves for steels with different Al concentrations during tensile tests until fracture at room temperature. The three austenitic steels (0Al, 1.5Al and 3Al) exhibit higher elongations but lower strength levels compared to the duplex steels (4.5Al, 6Al and 6Al12Ni). The results clearly indicate strengthening but ductility loss induced by the presence of ferrite. Given the high ductility of standard duplex stainless steels without B2 in ferrite [60,61], the pronounced ductility loss in the present case can be attributed to the presence of B2 intermetallics within ferrite [62].

The austenitic microstructure of 0Al, 1.5Al and 3Al steels enables to study the influence of Al on the work hardening response of austenite. Given the comparable C concentration of 0Al and 3Al alloys and the large difference in their Al concentration, the work hardening behavior is compared for these two steels only. The strain dependence of true work hardening rate for 0Al and 3Al alloys is plotted in Figure 10b. According to Figure 10b, the work hardening rate at low strains is higher for the 3Al steel. The initially high work hardening rate of 3Al steel gradually decreases at higher strains. The 0Al steel, on the other hand, exhibits an almost constant work hardening rate until necking. As a result, the work hardening curves cross at a true strain of 0.21. The observation of an initially higher work hardening rate for the 3Al steel does not agree with the well-documented effect of Al as an SFE raiser and promoter of wavy glide [15,26,63,64] and the observations made for a similar stainless-steel system in wrought condition [4]. Each wt.% of Al has been reported to increase the SFE of austenitic steels by nearly 8.8 mJ/m^2^ [65]. If the empirical relationship proposed by Qi-Xun et al. [66] is used for a rough estimation of SFE and the effect of Al on the SFE is accounted for by the preceding factor, SFEs for 0Al, 1.5Al and 3Al steels are estimated to be of the order of 33 mJ/m^2^, 49 mJ/m^2^ and 58 mJ/m^2^, respectively.

**Figure 11:**
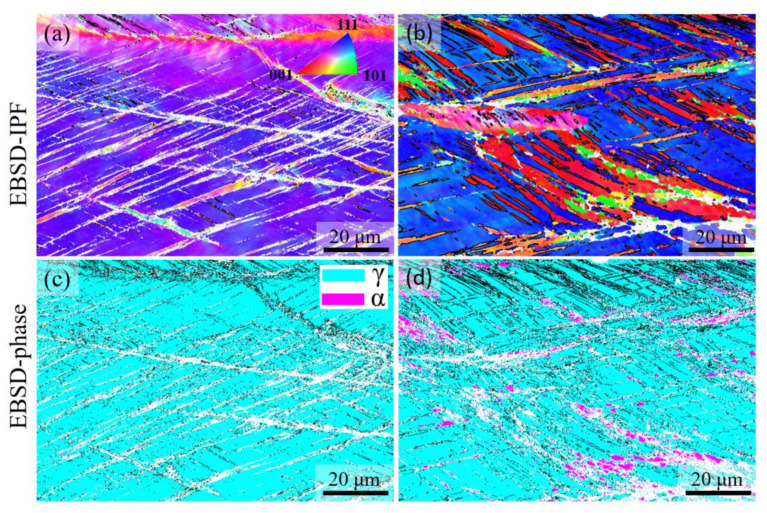
EBSD results for (**a**,**c**) 0Al and (**b**,**d**) 3Al steels after tensile deformation until fracture at room temperature. (**a**,**b**) IPF maps, (**c**,**d**) phase maps. Colors in IPF maps indicate crystal directions parallel to the tensile direction. In (**a**,**b**), boundaries within ±5° from the Σ3 twin relationship are superimposed using black lines.

To identify the deformation mechanisms and clarify the origin of initially high work hardening rates in the 3Al steel, post-mortem microstructural examinations by EBSD were conducted for 0Al and 3Al steels. Figure 11a,b present the IPF maps with Σ3 twin boundaries superimposed in black. The corresponding phase maps are shown in Figure 11c,d. According to the EBSD IPF maps, twins are clearly induced in both alloys, even though Al increases the SFE and the critical stress for twinning [67]. Therefore, the SFE increase due to Al addition has not been significant enough to suppress the formation of twins. Deformation twinning has also been reported after room temperature tensile deformation of Fe-17Cr-9Ni-7Al-6Mn-0.46C (wt.%) [47], even though the empirical relationship proposed by Qi-Xun et al. [68]—coupled with the factor proposed by Jeong et al. [65] for the effect of Al—returns a high SFE of 105 mJ/m^2^. Given the orientation dependence of twinning [68], the limited spatial resolution of EBSD in detecting fine twins [69], and low EBSD pattern qualities in the case of highly deformed microstructures [70], no effort was made to quantify and compare deformation twin fractions in 0Al and 3Al steels. Nevertheless, continuous fragmentation of austenite grains by deformation twins, also referred to as the dynamic Hall-Petch effect [71], is expected to have contributed to the work hardening of both alloys [72].

The EBSD phase maps in Figure 11c,d indicate the presence of a small fraction of deformation-induced martensite in the case of the 3Al steel. This is not compatible with the higher SFE of the 3Al steel and the observation of a reduced M_d_^γ→α‘^ temperature upon Al addition to a similar stainless-steel system in the wrought condition [4]. The occurrence of deformation-induced martensite is a likely mechanism responsible for the initially high work hardening rate of the 3Al steel. A closer inspection of the spatial distribution of regions having undergone deformation-induced martensitic transformation indicated that the transformation was confined to the dendrite cores and did not extend into interdendritic regions. On this basis, the ferritic solidification of the 3Al steel influenced the segregation pattern of alloying elements in such a manner that the SFE of ferritic dendrite cores became low enough to trigger martensitic transformation, even though these regions are enriched with Al. Although the solidification segregation was weakened by homogenization heat treatment, it is not eliminated fully. In the case of an austenitic stainless steel with pronounced segregation of alloying elements, the localized formation of martensite within the dendrite cores has been clearly demonstrated [73]. In such cases, the work hardening rate from the tensile test can be regarded as the average for regions with different SFEs. In the present case, the high glide planarity of the low-SFE regions near dendrite cores and the associated occurrence of deformation-induced martensitic transformation is expected to have contributed to the initially high work hardening rate of the 3Al steel. The strengthening of these regions will then cause deformation propagation to the surrounding high-SFE interdendritic regions, which, in turn, decreases the work hardening rate.

As an alternative or complementary to the preceding mechanism, the initially high work hardening rate of the 3Al steel could be related to the presence of LAGBs, as presented in Figure 6c,f. Although such boundaries are not as effective as HAGBs against the motion of dislocations [48], they could still serve as barriers, thereby supplementing the effect of HAGBs. The efficiency of LAGBs would, however, gradually diminish at higher strains as planar glide features, especially deformation twin boundaries, replace grain boundaries as dominant barriers regulating the mean free path of dislocations. The observation of an initially higher work hardening rate in an Al-added austenitic stainless steel containing LAGBs, but too stable to undergo deformation-induced martensite formation during tensile deformation at room temperature [14,48], lends support to the possibility of LAGBs being responsible for the behavior observed in the present case.

### 3.6. Fractography

**Figure 12:**
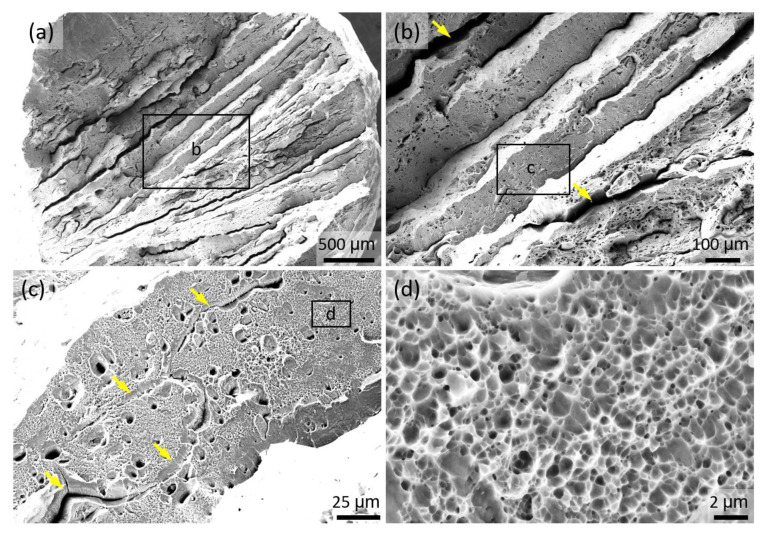
Fracture surface of 3Al steel at different magnifications. (**a**) Overview of the fracture surface; (**b**–**d**) magnified view of the regions marked by rectangles, see the label within each rectangle to locate. Arrows in (**b**,**c**) indicate decohesion along grain and subgrain boundaries, respectively. (**d**) provides a magnified view of dimples in the austenite.

Fracture surface examinations after tensile tests indicated a fairly similar appearance for all three austenitic steels. In Figure 12, the fracture surface for a tensile-tested specimen of 3Al steel is shown as representative for the austenitic steels. According to the low-magnification images in Figure 12a,b, the topology of the fracture surface is strongly influenced by the outline of dendrites and grain boundaries. Decohesion perpendicular to the plane of view (parallel to loading direction), as marked by arrows in Figure 12b, indicates the development of high tensile stresses perpendicular to the tensile loading axis. Since the glide systems activated in each grain are decided by their initial orientation and vary across grain boundaries, the shape change caused by the activation of slip systems varies from one grain to another. In the case of the present materials with coarse grain sizes, the density of geometrically necessary dislocations and the magnitude of stresses at grain boundaries can become high enough to induce decohesion and tunneling parallel to the loading axis. In the case of the 3Al steel, fractography also revealed light decohesion along some of the subgrain boundaries, as exemplified by arrows in Figure 12c. Otherwise, the occurrence of dimples characteristic of ductile fracture was the most obvious feature of the fracture surface for the austenitic alloys (Figure 12d).

**Figure 13:**
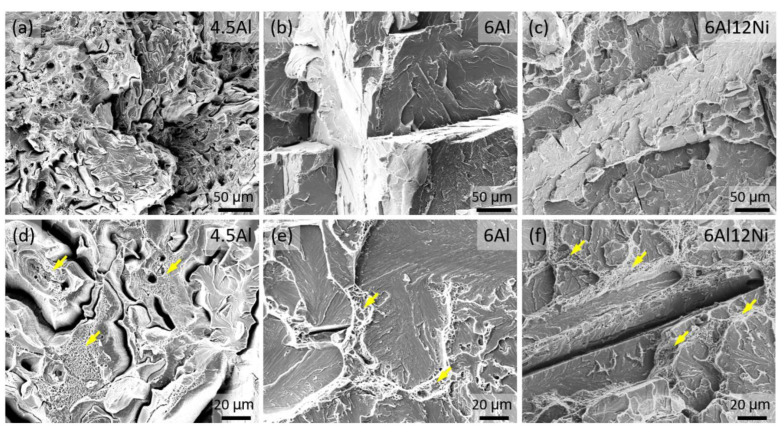
Fracture surface of (**a**,**d**) 4.5Al steel, (**b**,**e**) 6Al steel and (**c**,**f**) 6Al12Ni steel. Arrows in (**d**–**f**) indicate regions exhibiting dimple formation.

Figure 13 presents fractographs of 4.5Al, 6Al and 6Al12Ni duplex stainless steels at two different magnifications. In all cases, the fracture surface consists of regions exhibiting both ductile and brittle fracture. The fraction of regions exhibiting ductile fracture with characteristic dimples, as marked by arrows in Figure 13d–f, is proportional to the fraction of austenite. Accordingly, fracture surface of the 4.5Al steel with the highest austenite fraction of duplex steels consisted of a large proportion of dimpled areas. The remainder of fracture surface for the 4.5Al steel consisted of dimple-free areas characteristic of cleavage fracture, as, for instance, visible to the right of Figure 13d. Regions with dimple formation and cleavage fracture are interpreted as austenite and ferrite, respectively. In the case of the 6Al steel with the lowest austenite fraction of duplex steels, on the other hand, the majority of the fracture surface exhibits cleavage fracture features with obvious terrace steps. Given that planes are the common cleavage planes in body-centered cubic materials [74,75], including Fe-Al binary steels [75], the terraces most readily visible in Figure 13b are thought to correspond to planes in ferrite. In the case of the 6Al12Ni steel, a mix of cleavage fracture in ferrite with numerous well-aligned cracks (Figure 13c) and dimples in austenite (arrows in Figure 13f) were observed. Given that standard duplex stainless steels are immune to cleavage fracture after conventional processing [60,61], cleavage in ferrite in the present duplex steels is attributed to the presence of Al. This susceptibility is likely caused by the formation of B2 precipitates or the clustering of Al and Ni in the early stages of B2 formation.

## 4. Conclusions

The influence of Al on the density, microstructure, and mechanical properties of Fe-12Cr-9Ni-3Mn-0.3C-xAl (x = 0.1–6 wt.%) steels, as well as a Fe-12Cr-12Ni-3Mn-0.3C-6Al steel, was studied. The following conclusions were drawn:

(1) Al addition changes the solidification mode from austenitic to ferritic-austenitic. After heat treatment at 1150 °C followed by quenching, the microstructure becomes almost fully austenitic for steels containing up to 3 wt.% Al and duplex for steels with higher Al contents. The fraction of ferrite in duplex steels increases with the Al content. In contrast to the primary austenite formed directly from the liquid phase, the austenite formed from primary ferrite contains low-angle boundaries.
(2) Al addition decreases the density by approximately 1.45% per 1 wt.% Al. A share of nearly 31% arises from the lattice dilatation, the rest from the substitution of heavier atoms by Al.
(3) Al addition only has a mild influence on the hardness of austenite. A dramatic increase in hardness is observed as the microstructure evolves to duplex. The noticeable hardening caused by the presence of ferrite is due to the formation of B2-(Ni,Fe)Al intermetallics in ferrite. The existence of B2 in ferrite was confirmed by SEM examinations and HE-XRD measurements.
(4) Tensile tests indicate hardening without a noticeable change in ductility as the Al concentration is increased to 3 wt.%. The work hardening rate of the austenitic steel containing 3 wt.% Al is initially higher than that of its Al-free counterpart. At higher strains, however, the relative values of work hardening rates are reversed. The initially high work hardening rate of the 3Al steel is correlated with the existence of LAGBs. An additional likely mechanism is the occurrence of a small fraction of deformation-induced martensite in the less stable microstructural regions. For steels with duplex microstructures, the yield and ultimate tensile strengths are higher than the austenitic steels and correlate with the ferrite content. Strengthening occurred at the expense of ductility. Fracture surface examinations after tensile tests revealed dimple formation in austenite and cleavage fracture in ferrite, the latter being responsible for the ductility loss in the presence of high ferrite contents.

## Figures and Tables

**Table 1:** Alloy IDs and chemical compositions (wt.%).

Alloy ID	Color	C	Al	Cr	Ni	Mn	Fe
0Al	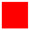	0.30	0.1	12.2	9.2	3.0	bal.
1.5Al	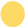	0.35	1.5	12.5	9.3	2.9	bal.
3Al	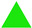	0.30	2.9	11.8	8.8	3.1	bal.
4.5Al	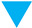	0.31	4.3	12.1	9.0	3.0	bal.
6Al	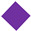	0.32	5.8	12.4	9.2	3.0	bal.
6Al12Ni	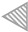	0.35	5.6	11.5	12.0	3.4	bal.

**Table 2:** Solidification modes based on thermodynamic equilibrium calculations by Thermo-Calc and DSC measurements. A and FA stand for austenitic and ferritic-austenitic solidification modes, respectively. In the latter case, the solidification begins with the formation of primary ferrite and continues with the formation of austenite.

Assessment Method	0Al	1.5Al	3Al	4.5Al	6Al
**Thermo-Calc**	A	FA	FA	FA	FA
**DSC**	A	FA	FA	FA	FA

## Data Availability

The raw/processed data required to reproduce these findings cannot be shared at this time due to technical or time limitations but are available from the corresponding author upon reasonable request.
